# Benzoxazolone Carboxamides: Potent and Systemically Active Inhibitors of Intracellular Acid Ceramidase**

**DOI:** 10.1002/anie.201409042

**Published:** 2014-11-13

**Authors:** Daniela Pizzirani, Anders Bach, Natalia Realini, Andrea Armirotti, Luisa Mengatto, Inga Bauer, Stefania Girotto, Chiara Pagliuca, Marco De Vivo, Maria Summa, Alison Ribeiro, Daniele Piomelli

**Affiliations:** Drug Discovery and Development, Istituto Italiano di TecnologiaVia Morego 30, 16163 Genova (Italy); Departments of Anatomy and Neurobiology, Pharmacology and Biological ChemistryUniversity of California Irvine, Irvine, CA 92697 (USA)

**Keywords:** acid ceramidase, cancer, ceramide, enzyme inhibition, sphingosine-1-phosphate

## Abstract

The ceramides are a family of bioactive lipid-derived messengers involved in the control of cellular senescence, inflammation, and apoptosis. Ceramide hydrolysis by acid ceramidase (AC) stops the biological activity of these substances and influences survival and function of normal and neoplastic cells. Because of its central role in the ceramide metabolism, AC may offer a novel molecular target in disorders with dysfunctional ceramide-mediated signaling. Here, a class of benzoxazolone carboxamides is identified as the first potent and systemically active inhibitors of AC. Prototype members of this class inhibit AC with low nanomolar potency by covalent binding to the catalytic cysteine. Their metabolic stability and high in vivo efficacy suggest that these compounds may be used as probes to investigate the roles of ceramide in health and disease, and that this scaffold may represent a promising starting point for the development of novel therapeutic agents.

The sphingolipids are a class of bioactive lipid molecules that serve multiple regulatory functions.[1] They contribute to key cellular processes[2] and are involved in the pathogenesis of inflammation and neuropathic pain.[3] The ceramides, a highly heterogeneous family of *N*-acylated sphingosines with long-chain fatty acids, hold a central position in sphingolipid metabolism[4] and have attracted considerable attention because of their proposed participation in cellular senescence,[5] inflammation,[6] and apoptosis.[7] Additionally, the ceramides are metabolic precursors of sphingosine-1-phosphate (S1P), a lipid mediator that enhances cell survival and proliferation by activating selective G protein-coupled receptors.[8] The pharmacology of sphingolipid signaling is still nascent, yet small-molecule modulators of ceramide production and degradation might open new avenues of therapeutic intervention in pathological conditions, including cancer, inflammation, and pain.[9]

AC is a lysosomal cysteine amidase that catalyzes the hydrolysis of ceramide into sphingosine and fatty acid (Figure 1). As this reaction is a crucial step in ceramide degradation and S1P biosynthesis,[10] AC inhibition can impact the balance between cell proliferation and death, influencing both growth and survival of normal and neoplastic cells.

**Figure 1:**
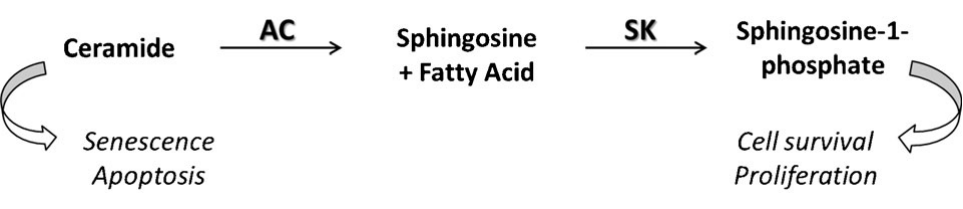
A simplified overview of the biosynthetic pathway leading from ceramide to sphingosine-1-phosphate. AC: acid ceramidase. SK: sphingosine kinase.

Although several AC inhibitors have been reported, the majority of these compounds are structural analogues of ceramide and are limited in their use by low inhibitory potency and/or lack of systemic activity (Figure 2, and Table S1 in the Supporting Information).[11,12]

**Figure 2:**
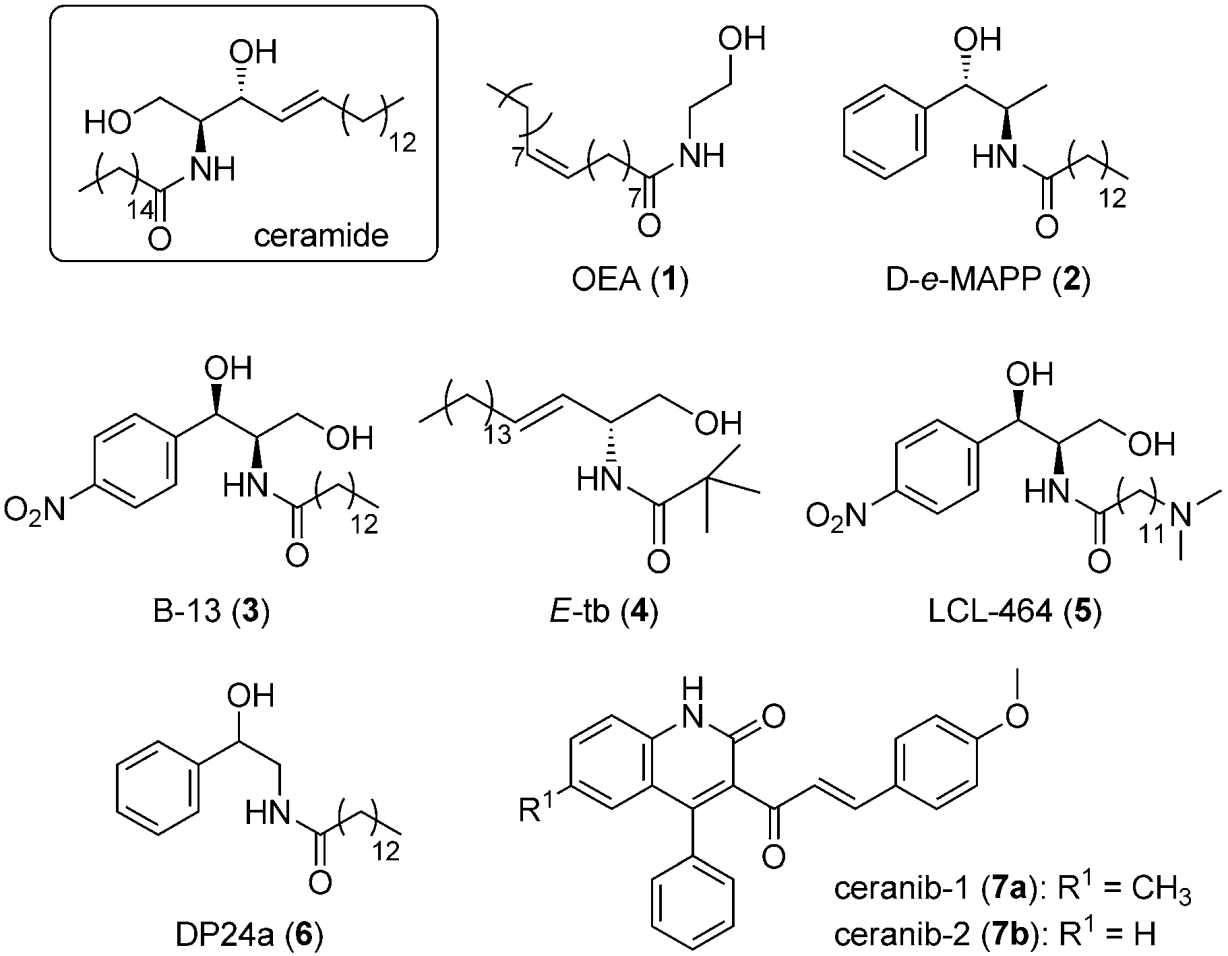
Structures of a representative ceramide species (d18:1/16:0) and various AC inhibitors (1–7).[13] OEA: oleoylethanolamide.

We previously identified the antineoplastic drug carmofur (5-fluoro-*N*-hexyl-2,4-dioxo-pyrimidine-1-carboxamide, **8**; Figure 3) as the first nanomolar inhibitor of AC (median inhibitory concentration, IC_50_, for rat AC=29 nm).[14] This result prompted us to expand the class of 2,4-dioxopyrimidine-1-carboxamide derivatives and led us to discover the first single-digit nanomolar inhibitors of AC activity.[15] Importantly, selected compounds in this series sensitize certain types of cancer cells to the actions of cytotoxic agents, such as 5-fluorouracil and taxol, which is suggestive of a potential clinical use as chemosensitizers. Despite their considerable potency, the utility of these agents is hindered by low chemical and metabolic stability. Even the best compounds in this class have extremely short half-life times in mouse plasma (*t*_1/2_=1–3 min),[14] a limitation that extensive structure–stability studies have been as yet unable to overcome.[15] Thus, the field still needs suitable small molecules to foster probe and drug discovery efforts.

**Figure 3:**
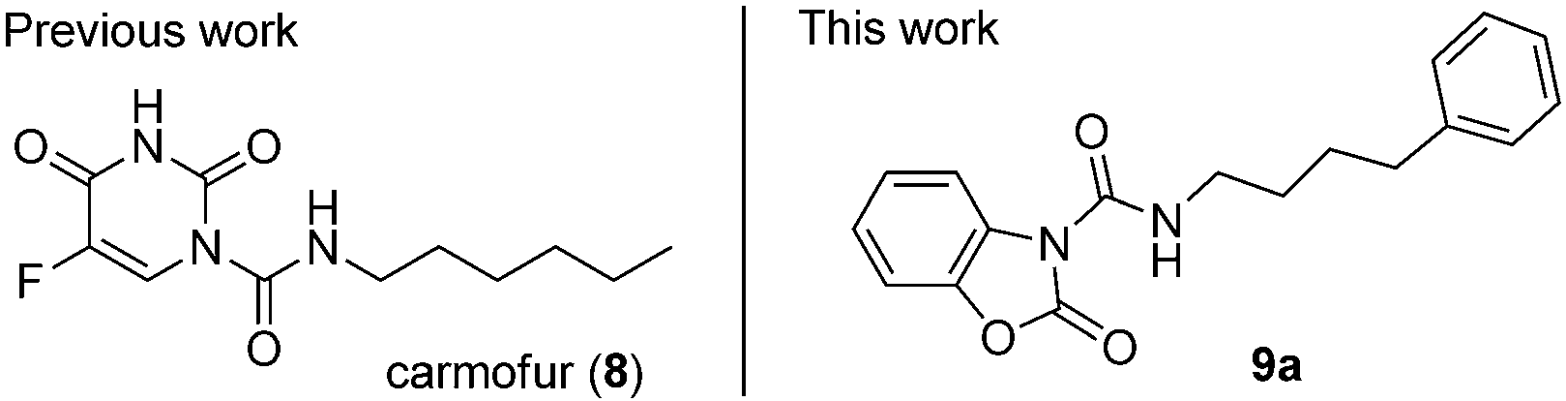
Initial hits used for the discovery of nanomolar AC inhibitors.

Here, we outline the discovery of the first class of potent AC inhibitors endowed with pronounced systemic activity. Elucidation of the mechanism through which these molecules inhibit AC along with in vivo pharmacokinetic and pharmocodynamic studies support the potential of this chemotype for the development of agents that target AC.

During the screening on human AC of a small library of compounds generated in our laboratory, we identified 2-oxo-*N*-(4-phenylbutyl)-1,3-benzoxazole-3-carboxamide (**9 a**) as a nanomolar inhibitor of this enzyme (IC_50_ for human AC=64 nm; Figure 3). Kinetic studies using human recombinant AC showed that **9 a** causes a concentration-dependent reduction in maximal catalytic velocity (*V*_max_) without affecting the Michaelis–Menten constant (*K*_m_; Figure 4).

**Figure 4:**
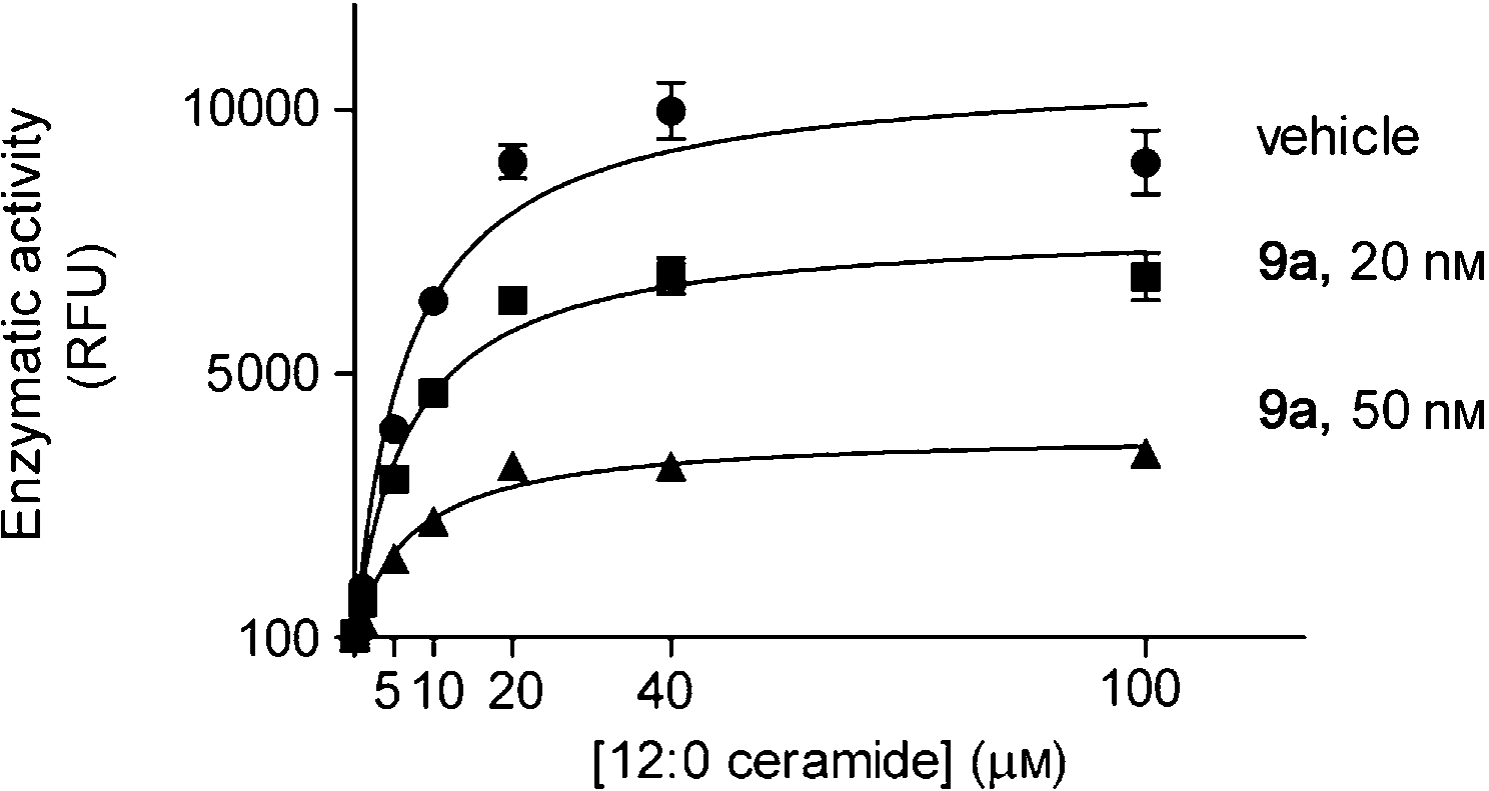
Kinetic analysis of the AC reaction in the presence of vehicle (DMSO 1 %, *n*=6) or 9 a (20 nm or 50 nm, *n*=6). *V*_max_ (vehicle)=10 793 RFU; Vmax (9 a, 20 nm)=7808 RFU; *V*_max_ (9 a, 50 nm)=3889 RFU. *K*_m_ (vehicle)=6.8 μm; *K*_m_ (9 a, 20 nm)=6.8 μm; *K*_m_ (9 a, 50 nm)=7.2 μm. RFU=relative fluorescence units. Data are expressed as mean of two independent experiments.

This result, which is suggestive of a noncompetitive mechanism of action, was extended by liquid chromatography mass spectrometry (LC-MS) experiments, which showed that **9 a** covalently binds to the catalytic cysteine, Cys-143, of the enzyme.[16] Because **9 a** contains two electrophilic carbonyl groups, it may form distinct adducts with AC. As illustrated in Figure 5 A, the nucleophilic attack of Cys-143 on the carbonyl group of urea would result in adducts **A** or **B**, whereas attack on the carbonyl group of the carbamate would result in adducts **C** or **D**. Incubation of purified recombinant human AC with **9 a**, followed by trypsin digestion and peptide analysis by LC-MS, showed the presence of a covalent adduct of **9 a** with the N-terminal peptide of AC (CTSIVAEDK, Figure 5 B, red trace). Control incubations of AC with DMSO showed only the native unmodified peptide (Figure 5 B, black trace). Tandem MS analysis showed the formation of adduct **A** and indicated that the mass increase, corresponding to the carboxamide side chain of **9 a**, is carried by the N-terminal cysteine, confirming that this residue is the target of covalent binding (Figure 5 C). We found no evidence supporting alternative hypotheses and no adducts of **9 a** with other AC tryptic peptides. These results indicate that **9 a** inhibits human AC through *S*-acylation of catalytic Cys-143, with the benzoxazolone ring acting as the leaving group.

**Figure 5:**
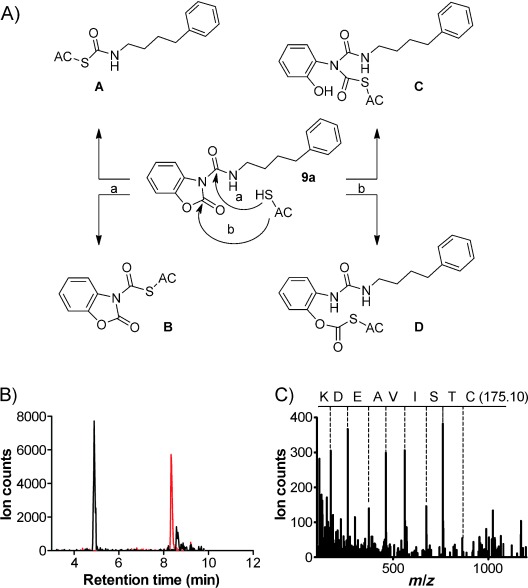
Compound 9 a inhibits AC by covalent modification of the catalytic Cys-143. Possible covalent adducts formed upon nucleophilic attack of AC catalytic Cys-143 on 9 a (A). Extracted-ion chromatograms of doubly-charged native peptide from a control incubation with DMSO (*m*/*z*: 483.23, black trace) and the same peptide covalently modified by 9 a (*m*/*z*: 570.79, red trace) (B). Tandem mass spectrum of the covalently modified peptide, confirming peptide sequence (CTSIVAEDK) and mass increase of Cys-143 (+175.10 Da); mass increase and peptide sequence are consistent with the formation of adduct A (+C_11_H_13_NO) (C).

Encouraged by the potency and drug-likeness of **9 a**, which is based on the privileged scaffold of benzoxazolone,[17] we sought to expand this chemical class and identify structural features that are critical for AC inhibition. Focused structure–activity relationship (SAR) studies were first aimed at exploring the role of the urea moiety of **9 a**, with a small set of compounds (**9 b**–**e**) that were prepared as described in Scheme 1.

**Scheme 1:**
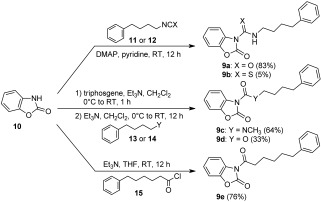
Syntheses of benzoxazolone derivatives 9 a–e.

Replacement of the carbonylic oxygen atom of the urea moiety of **9 a** with a sulfur atom gave compound **9 b**. This derivative showed limited chemical stability in [D_6_]DMSO, and was not studied further. The role of the 3-carboxamide NH group was then investigated by preparing the *N*-methyl derivative **9 c** and the corresponding carbamate **9 d**. The two compounds were efficiently accessed by activating the benzoxazolone **10** with triphosgene, followed by in situ treatment with the appropriate commercial amine **13** or alcohol **14**, respectively. Both compounds **9 c** and **9 d** had no inhibitory activity on AC. Loss of activity was also observed when the urea moiety was replaced with an amide, as in compound **9 e**. These results demonstrate that the 3-carboxamide NH moiety is a key structural feature for AC inhibition (Table 1, entries 1–5). We reasoned then that we could modulate the reactivity of the 3-carboxamide by introducing substituents with different electronic properties on the heterocyclic scaffold. To test this hypothesis, we prepared compounds **16 a** and **17 a**, which bear a bromine and a *p*-fluorophenyl group at position 6, respectively. Compound **16 a** was obtained by coupling the commercial 6-bromobenzoxazolone **18** to 4-phenylbutyl isocyanate **11**, while **17 a** was accessed by a two-step synthesis involving a Suzuki–Miyaura reaction to give **19**, followed by N3 acylation (Scheme 2).

**Scheme 2:**
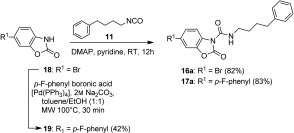
Syntheses of substituted benzoxazolone derivatives 16 a and 17 a.

**Table 1:** Inhibitory potencies (IC_50_) of compound 9 a, analogues 9 b–9 e, and 16 a-17 a on human AC. 
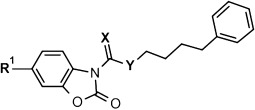

Entry	Compound	R^1^	X	Y	IC_50_ [nm][a] ±S.E.M
1	**9 a**	H	O	NH	64±7
2	**9 b**	H	S	NH	not stable
3	**9 c**	H	O	NCH_3_	no inhibition
4	**9 d**	H	O	O	no inhibition
5	**9 e**	H	O	CH_2_	no inhibition
6	**16 a**	Br	O	NH	31±9
7	**17 a**	*p*-F-Phe	O	NH	79±31

[a] IC_50_ values are expressed as means of at least three determinations.

The introduction of a bromine in the *para* position to the urea moiety, as in compound **16 a**, led to a two-fold increase in potency relative to **9 a** (**16 a**, IC_50_=33 nm), while replacement of bromine with *p*-fluorophenyl had no significant effect (**17 a**, IC_50_=79 nm; Table 1, entries 6–7).

We expected that modulating the reactivity of these compounds would impact their stability. Therefore, we tested derivatives **9 a**, **16 a**, and **17 a** for their stability in buffers at acidic and neutral pH values, as well as in mouse plasma (Table 2). All compounds showed adequate stability under standard assay conditions (pH 4.5). When tested in phosphate-buffered saline (PBS, pH 7.4), the introduction of bromine in the *para* position to the urea moiety, as in compound **16 a**, led to a decrease of stability compared to **9 a** (**16 a**, *t*_1/2_=24 min), whereas replacement of bromine with a *p*-fluorophenyl group promoted stability (**17 a**, *t*_1/2_>300 min). As expected, an electron-withdrawing group increased the electrophilicity of the carbonyl group of urea and made the resulting benzoxazolone a better leaving group upon nucleophilic attack, accounting for the lower stability observed in neutral buffer. Interestingly, the highly conjugated system resulting from the introduction of the phenyl ring, as in compound **17 a**, stabilizes the benzoxazolone 3-carboxamide scaffold and, at the same time, appears to be well tolerated in terms of AC inhibitory potency. Stability experiments in mouse plasma showed that **17 a** has a substantially longer plasma half-life than does **9 a** (**17 a**, *t*_1/2_>120 min) and is considerably more stable than the corresponding bromine derivative **16 a** (Table 2).

**Table 2:** Stability of compounds 9 a and 16 a–17 a by LC-MS analysis.

Entry	Compound	Buffer stability[a] (pH 4.5) *t*_1/2_ [min]	Buffer stability[b] (pH 7.4) *t*_1/2_ [min]	*m*-Plasma stability[c] *t*_1/2_ [min]
1	**9 a**	126±3	45	60
2	**16 a**	294±30	24	8
3	**17 a**	>360	>300	>120

[a] NaCl (150 mm), NaH_2_PO_4_ (100 mm), trisodium citrate (100 mm), NP40 (1 %), DTT (3 mm).

[b] PBS.

[c] Mouse plasma, 37 °C.

Furthermore, metabolic stability studies in mouse liver microsomes showed that 89 % of **17 a** was recovered after an incubation time of one hour. Lastly, compound **17 a** was tested for off-target effects on a set of enzymes that includes proteases (aspartic, cysteine, and serine), lipoxygenases, cyclooxygenases, group IV phospholipase (sPLA_2_), and monoacylglycerol lipase. The compound showed no significant activity toward these targets, with the exception of a weak inhibitory effect on the aspartic protease cathepsin D (67 % inhibition at 10 μm; Table S2, Supporting Information).

The favorable profile of **17 a** prompted us to test its ability to inhibit AC in intact cells. Human colon adenocarcinoma SW403 cells and mouse macrophage-like Raw 264.7 cells were incubated in the presence of **17 a** (0.1–20 μm). AC activity and sphingolipid levels were measured after various incubation times. The compound inhibited cellular AC activity with an IC_50_ of 825 nm in SW403 and 400 nm in Raw 264.7 cells (Figure 6 A,B). Consistent with these results, incubation with **17 a** resulted in an increase in the levels of ceramide (d18:1/16:0) and a corresponding decrease in the levels of sphingosine. The levels of dihydroceramide (d18:0/16:0), which is cleaved by AC to sphinganine,[1b] were also increased (Figure 6 C,D).

**Figure 6:**
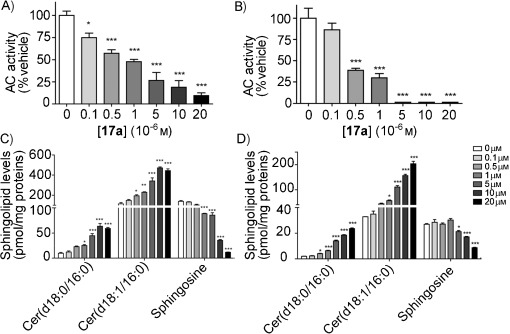
Effects of compound 17 a in SW403 (A, C) and Raw 264.7 cells (B, D), after a 3 h incubation. Concentration dependence of the effects on AC activity (A, B) and sphingolipid levels (C, D). Values are expressed as means ±S.E.M of at least three determinations. Experiments were repeated twice with similar results.

The effects of **17 a** persisted for 6 h, with a partial recovery of enzyme activity and consequent decrease in sphingolipid levels observed after 24 h (Figure 7). The results indicate that **17 a** inhibits AC in a complex cellular environment, leading to the intended biochemical response, that is, increased ceramide and decreased sphingosine levels.

**Figure 7:**
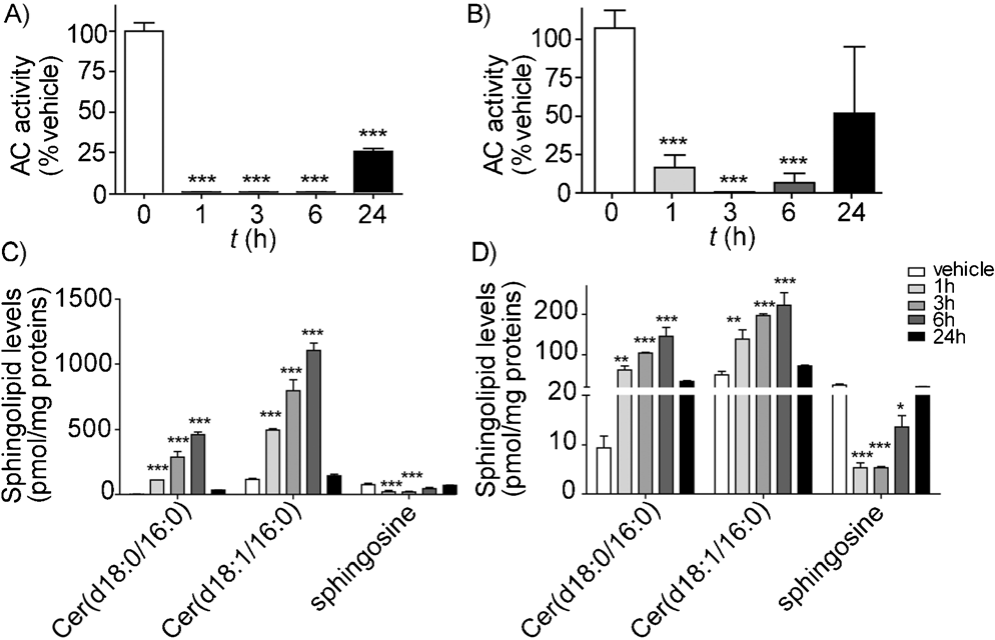
Time-course of the effects of 17 a (20 μm) in SW403 (A, C) and Raw 264.7 cells (B, D) on AC activity (A, B) and sphingolipid levels (C, D). Values are expressed as means ±S.E.M of at least three determinations. Experiments were repeated twice with similar results.

Pharmacokinetic analyses showed that **17 a** quickly enters the bloodstream after a single intraperitoneal (i.p. 10 mg kg^−1^) administration in mice (Figure 8 A), reaching a maximal plasma concentration, C_max_, of 1767.9 ng mL^−1^ and displaying a half-life time of 458 min in circulation. Relevant pharmacokinetic parameters are reported in Table S3 (Supporting Information). The primary in vivo metabolite of **17 a**, the hydrolysis product **19** (Figure 8 B), did not inhibit AC in vitro at 10 μmμm.

**Figure 8:**
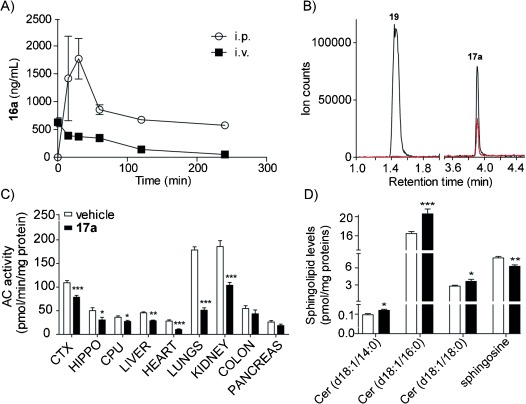
In vivo profile of 17 a. Plasma pharmacokinetic profile of 17 a after i.p. (10 mg kg^−1^) and i.v. (1 mg kg^−1^) administration in mice (A). Identification of 19 as primary in vivo metabolite of 17 a: superimposed MRM traces of a standard sample of 17 a (retention time 3.91 min, 1 μm calibrator, red trace) and a sample collected 1 h after i.p. administration of 17 a in mice (10 mg kg^−1^; black trace) (B). The peak at 1.4 min corresponds to the primary metabolite of 17 a (19, 227 Da molecular mass, *m*/*z*: 228 detected in ESI mode). Effects of 17 a (10 mg kg^−1^, 3 h) on AC activity in mouse tissues (C) and sphingolipid levels in lungs (D). Values are expressed as means ±S.E.M (*n*=6).

Injection of **17 a** in mice (10 mg kg^−1^, i.p.) caused a substantial reduction in AC activity in multiple organs, including brain, liver, heart, lungs, and kidney (Figure 8 C). Highest levels of baseline AC activity and AC inhibition were found in lung tissue, which was selected for further analyses. As expected, we observed a significant increase in ceramide species that are preferred substrates for AC (namely, d18:1/14:0, d18:1/16:0, and d18:1/18:0) along with a concomitant decrease in sphingosine (Figure 8 D). The levels of ceramides with longer fatty acyl chains, which are not preferred substrates for AC, were not affected (Table S4, Supporting Information). Compound **17 a** appeared to be well tolerated at the dosage tested. These findings show that **17 a** inhibits AC in live animals, resulting in the predicted alteration of the balance between ceramide and sphingosine.

In conclusion, we identified the first class of potent and systemically active inhibitors of intracellular AC activity. These compounds act as covalent inhibitors of AC. Focused SAR studies showed that an unsubstituted nitrogen atom in the 3-carboxamide moiety is mandatory for activity, and highlighted that the introduction of a *p*-fluorophenyl group on the benzoxazolone ring balanced potency and stability (both chemical and metabolic), as shown with compound **17 a**. Notably, these features are coupled with the ability of **17 a** to inhibit AC in various cell lines as well as in vivo. Thus, our results identify compound **17 a** as the first potent chemical probe that may be used to investigate the physiopathological roles of ceramide and as starting point for the discovery of novel therapeutic agents. Further expansion of this chemical class will be reported in due course.

## References

[1a] Hannun YA, Obeid LM (2008). Nat. Rev. Mol. Cell Biol.

[1b] Bartke N, Hannun YA (2009). J. Lipid Res.

[2a] Ogretmen B, Hannun YA (2004). Nat. Rev. Cancer.

[2b] Gangoiti P, Camacho L, Arana L, Ouro A, Granado MH, Brizuela L, Casas J, Fabrias G, Abad JL, Delgado A, Gomez-Munoz A (2010). Prog. Lipid Res.

[2c] Dimanche-Boitrel MT, Rebillard A (2013). Handb. Exp. Pharmacol.

[3a] Salvemini D, Doyle T, Kress M, Nicol G (2013). Trends Pharmacol. Sci.

[3b] Patti GJ, Yanes O, Shriver LP, Courade JP, Tautenhahn R, Manchester M, Siuzdak G (2012). Nat. Chem. Biol.

[4] Mao C, Obeid LM (2008). Biochim. Biophys. Acta Mol. Cell Biol. Lipids.

[5] Huang X, Withers BR, Dickson RC (2014). Biochim. Biophys. Acta Mol. Cell Biol. Lipids.

[6] Maceyka M, Spiegel S (2014). Nature.

[7a] Pettus BJ, Chalfant CE, Hannun YA (2002). Biochim. Biophys. Acta Mol. Cell Biol. Lipids.

[7b] Morales A, Lee H, Goni FM, Kolesnick R, Fernandez-Checa JC (2007). Apoptosis.

[7c] Huang WC, Chen CL, Lin YS, Lin CF (2011). J. Lipids.

[8a] Spiegel S, Milstien S (2003). Nat. Rev. Mol. Cell Biol.

[8b] Takabe K, Spiegel S (2014). J. Lipid Res.

[9a] Nussbaumer P (2008). ChemMedChem.

[9b] Canals D, Perry DM, Jenkins RW, Hannun YA (2011). Br. J. Pharmacol.

[9c] Adan-Gokbulut A, Kartal-Yandim M, Iskender G, Baran Y (2013). Curr. Med. Chem.

[9d] Truman J, Garcia-Barros M, Obeid LM, Hannun YA (2014). Biochim. Biophys. Acta Mol. Cell Biol. Lipids.

[10] Gault CR, Obeid LM, Hannun YA (2010). Adv. Exp. Med. Biol.

[11] Saied EM, Arenz C (2014). Cell. Physiol. Biochem.

[12a] Bielawska A, Greenberg MS, Perry D, Jayadev S, Shayman JA, McKay C, Hannun YA (1996). J. Biol. Chem.

[12b] Raisova M, Goltz G, Bektas M, Bielawska A, Riebeling C, Hossini AM, Eberle J, Hannun YA, Orfanos CE, Geilen CC (2002). FEBS Lett.

[12c] Bedia C, Canals D, Matabosch X, Harrak Y, Casas J, Llebaria A, Delgado A, Fabrias G (2008). Chem. Phys. Lipids.

[[12d[] Bai A, Szulc ZM, Bielawski J, Mayroo N, Liu X, Norris J, Hannun YA, Bielawska A (2009). Bioorg. Med. Chem.

[12e] Proksch D, Klein JJ, Arenz C (2011). J. Lipids.

[12f] Draper JM, Xia Z, Smith RA, Zhuang Y, Wang W, Smith CD (2011). Mol. Cancer Ther.

[13] See the Supporting Information (Table S1) for a summary of AC inhibitory activity data for compounds **1****7**

[14] Realini N, Solorzano C, Pagliuca C, Pizzirani D, Armirotti A, Luciani R, Costi MP, Bandiera T, Piomelli D (2013). Sci. Rep.

[15] Pizzirani D, Pagliuca C, Realini N, Branduardi D, Bottegoni G, Mor M, Bertozzi F, Scarpelli R, Piomelli D, Bandiera T (2013). J. Med. Chem.

[16] Shtraizent N, Eliyahu E, Park JH, He X, Shalgi R, Schuchman EH (2008). J. Biol. Chem.

[17] Poupaert J, Carato P, Colacino E, Yous S (2005). Curr. Med. Chem.

