# Supplementary material for: Benzoxazolone Carboxamides: Potent and Systemically Active Inhibitors of Intracellular Acid Ceramidase
Source: Angew Chem Int Ed Engl. 2014 Nov 13;54(2):485–9. doi: 10.1002/anie.201409042 (PMC4502975; doi:10.1002/anie.201409042)
Supplement: Supplementary file 1 [file anie0054-0485-sd1.pdf]

Angewandte Chemie
Eine Zeitschrift der Gesellschaft Deutscher Chemiker

Supporting Information

© Wiley-VCH 2014

69451 Weinheim, Germany

Benzoxazolone Carboxamides: Potent and Systemically Active Inhibitors of Intracellular Acid Ceramidase**

*Daniela Pizzirani, Anders Bach, Natalia Realini, Andrea Armirotti, Luisa Mengatto, Inga Bauer, Stefania Girotto, Chiara Pagliuca, Marco De Vivo, Maria Summa, Alison Ribeiro, and Daniele Piomelli**

anie_201409042_sm_miscellaneous_information.pdf

Supporting Information

Contents

1. General Information S2
2. Synthesis and Characterization of compounds **9a**-**e**, **16a**-**17a**, and **19** S3
3. Synthesis and Characterization of the Fluorogenic Probe S6
4. Summary of AC Inhibitory Activity Data for Literature Compounds (**1**-**7**) S7
5. Biology – Materials and Methods S7
6. Human AC expression and purification S10
7. Liquid Chromatography-Mass Spectrometry (LC-MS) Study S10
8. Analytical Stability Data S11
9. Mouse Liver Microsomal Stability S11
10. Off-Target Pharmacology S12
11. Pharmacokinetic Study S12
12. *Ex Vivo* Quantification of Long-Chain Ceramides S14
13. References S14

1. General Information

Chemicals, Materials and Methods. All the commercial available reagents and solvents were used as purchased from vendors without further purification. Dry solvents (pyridine, DCM) were purchased from Sigma-Aldrich. Automated column chromatography purifications were done using a Teledyne ISCO apparatus (CombiFlash® Rf) with pre-packed silica gel columns of different sizes (from 4 g up to 40 g). Mixtures of increasing polarity of cyclohexane and ethyl acetate (EtOAc) were used as eluents. Microwave heating was performed using Explorer®-48 positions instrument (CEM). NMR experiments were run on a Bruker Avance III 400 system (400.13 MHz for ^{1}H, and 100.62 MHz for ^{13}C), equipped with a BBI probe and Z-gradients. Spectra were acquired at 300 K, using deuterated dimethylsulfoxide (DMSO-d_6), deuterated chloroform ($CDCl_3$) as solvents. Chemical shifts for ^{1}H and ^{13}C spectra were recorded in parts per million using the residual non-deuterated solvent as the internal standard (for $CDCl_3$: 7.26 ppm, ^{1}H and 77.16 ppm, ^{13}C; for DMSO-d_6: 2.50 ppm, ^{1}H; 39.52 ppm, ^{13}C). LC-MS analyses were run on a Waters ACQUITY UPLC-MS system consisting of a SQD (Single Quadrupole Detector) Mass Spectrometer equipped with an Electrospray Ionization interface and a Photodiode Array Detector. PDA range was 210-400 nm. Analyses were performed on an ACQUITY UPLC HSS T3 C_{18} column (50 × 2.1 mm ID, particle size 1.8 µm) with a VanGuard HSS T3 C_{18} pre-column (5 × 2.1 mm ID, particle size 1.8 µm). Mobile phase was either 10 mM NH_4OAc in H_2O at pH 5 adjusted with AcOH (A) and 10 mM NH_4OAc in MeCN-H_2O (95:5) at pH 5 (B). Electrospray ionization in positive and negative mode was applied. All intermediates, and final compounds **9a**-**e** and **16a-17a** showed ≥ 95% purity by NMR (^{1}H, ^{13}C, ^{1}H-^{1}H COSY, ^{1}H-^{13}C HSQC) and UPLC-MS (UV). DMSO stock solutions of final compounds (10 mM) used for biological tests were evaluated prior to tests (NMR, LC-MS), and concentration was assessed by quantitative ^{1}H-NMR.

Abbreviations. Acetonitrile (MeCN), ammonium carbonate ($(NH_4)_2CO_3$), ammonium chloride (NH_4Cl), carbon dioxide (CO_2), dichloromethane (DCM), 4-(dimethylamino)-pyridine (DMAP), dimethylformamide (DMF), dimethylsulfoxide (DMSO), ethanol (EtOH), ethyl acetate (EtOAc), 1-ethyl-3-(3-dimethylaminopropyl)carbodiimide hydrochloride (EDC·HCl), magnesium chloride ($MgCl_2$), methanol (MeOH), sodium carbonate (Na_2CO_3), sodium hydroxide (NaOH), sodium periodate ($NaIO_4$), sodium sulfate (Na_2SO_4), tetrahydrofuran (THF), tetrakis(triphenylphosphine) palladium(0) ($Pd(PPh_3)_4$), thionyl chloride ($SOCl_2$), triethylamine (Et_3N). Other abbreviations used are: aqueous (aq.), hours (h), minutes (min), room temperature (rt), saturated (sat.).

2. Synthesis and Characterization of 9a-9e, 16a-17a, and 19

2-Oxo-*N*-(4-phenylbutyl)-1,3-benzoxazole-3(2*H*)-carboxamide (9a)

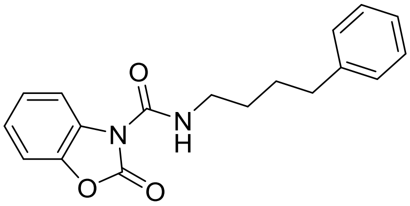

3*H*-1,3-Benzoxazol-2-one **10** (50 mg, 0.37 mmol) was dissolved in dry pyridine (2.2 mL). DMAP (50.1 mg, 0.41 mmol) was added and the reaction mixture stirred under nitrogen atmosphere at rt for 30 min. 4-Phenylbutyl isocyanate **11** (104 mg, 0.100 mL, 0.59 mmol) was added and the resulting mixture was stirred for 15 h. The solvent was removed under reduced pressure, and the compound was purified by silica gel column chromatography (cyclohexane:EtOAc 70:30) to afford **9a** as a white solid (95 mg, 83%). **^{1}H NMR** (400 MHz, DMSO-d_6) δ 8.14 (t, *J*=5.7 Hz, 1H), 7.92 – 7.86 (m, 1H), 7.45 – 7.38 (m, 1H), 7.31 – 7.23 (m, 5H), 7.23 – 7.19 (m, 2H), 7.19 – 7.13 (m, 1H), 3.39 – 3.32 (m, 2H), 2.61 (t, *J*=7.3 Hz, 2H), 1.71 – 1.51 (m, 4H). **^{13}C NMR** (101 MHz, DMSO-d_6) δ 152.1, 149.3, 142.0, 141.5, 128.3, 128.2, 125.6, 124.5, 124.1, 114.5, 109.9, 39.4, 34.7, 28.6, 28.1. **MS** (ESI) *m/z*: 311 [M + H]$^+$.

2-Oxo-*N*-(4-phenylbutyl)benzoxazole-3(2*H*)-carbothioamide (9b)

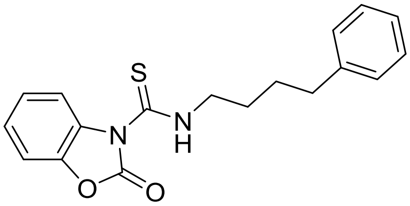

Compound **9b** was obtained according to the procedure previously described for **9a**, starting from 3*H*-1,3-benzoxazol-2-one **10** (100 mg, 0.467 mmol) and 4-phenylbutyl isothiocyanate **12** (90 mg, 0.088 mL, 0.514 mmol). After 2 h, another portion of **12** (90 mg) was added, and the reaction was stirred for 18 h. The crude was purified by silica gel column chromatography (cyclohexane:EtOAc 85:15 to 80:20) to afford **9b** a white solid (11.3 mg, 5%). **^{1}H NMR** (400 MHz, $CDCl_3$) δ 10.17 (br s, 1H), 8.99 – 8.92 (m, 1H), 7.31 – 7.23 (m, 5H), 7.21 – 7.16 (m, 3H), 3.81 – 3.74 (m, 2H), 2.70 (t, *J*=7.0 Hz, 2H), 1.82 – 1.75 (m, 4H). **^{13}C NMR** (101 MHz, $CDCl_3$) δ 176.4, 153.0, 141.9, 141.8, 129.2, 128.5, 126.1, 125.1, 124.5, 118.0, 110.0, 45.4, 35.5, 28.8, 27.5. **MS** (ESI) *m/z*: 327 [M + H]$^+$.

***N*-Methyl-2-oxo-*N*-(4-phenylbutyl)benzoxazole-3(2*H*)-carboxamide (9c)**

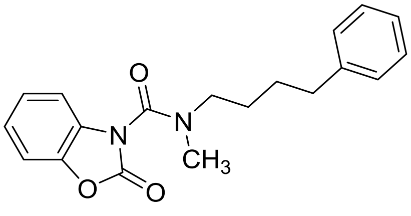

Triphosgene (110 mg, 0.37 mmol) was dissolved in dry DCM (3.7 mL) under nitrogen atmosphere. A solution of 3*H*-1,3-benzoxazol-2-one **10** (50 mg, 0.37 mmol) and Et_3N (0.29 mL, 2.08 mmol) in dry DCM (1.0 mL) was added at 0 °C and the reaction mixture was stirred for 1 h. Then, the reaction was cooled back to 0 °C and a solution of 4-phenylbutyl methylamine hydrochloride **13** (90 mg, 0.55 mmol) and Et_3N (0.08 mL, 0.55 mmol) in dry DCM (1.0 mL) was added. The resulting solution was allowed to warm to rt and stirred for 12 h. The reaction mixture was diluted with DCM (5 mL) and quenched with sat. aq. NH_4Cl (5 mL). The two layers were separated and the aqueous phase was extracted with DCM (3 × 10 mL). The combined organic layer was dried over Na_2SO_4, filtered, concentrated and purified by silica gel column chromatography (cyclohexane:EtOAc 85:15) to afford **9c** as a colorless oil (77 mg, 64%). **^{1}H NMR** (400 MHz, $CDCl_3$) δ 7.31 – 7.26 (m, 4H), 7.24 – 7.15 (m, 8H), 3.74 – 3.34 (m, 2H), 3.09 (s, 3H), 2.78 – 2.47 (m, 2H), 1.84 – 1.60 (m, 4H). **^{13}C NMR** (101 MHz, $CDCl_3$) δ 28.2, 35.5, 36.6, 49.7, 110.2, 112.8, 124.0, 124.4, 126.0, 128.5, 129.2, 142.0, 143.1, 150.0, 150.6. **MS** (ESI) *m/z*: 325 $[M + H]^+$, 342 $[M + NH_4]^+$.

4-Phenylbutyl 2-oxobenzoxazole-3(2*H*)-carboxylate (9d)

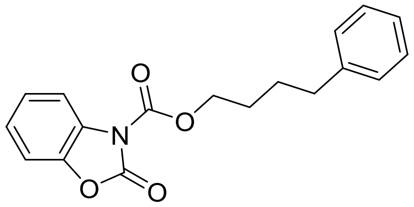

Compound **9d** was prepared according to the procedure previously described for **9c**, starting from 3*H*-1,3-benzoxazol-2-one **10** (70 mg, 0.52 mmol) and 1-phenyl-4-butanol **14** (117 mg, 0.120 mL, 0.78 mmol). The crude was purified by silica gel column chromatography (cyclohexane:EtOAc 92:8) to afford **9d** as a white powder (53 mg, 33%). **^{1}H NMR** (400 MHz, $CDCl_3$) δ 7.91 – 7.60 (m, 1H), 7.32 – 7.26 (m, 2H), 7.26 – 7.16 (m, 6H), 4.49 (t, *J*=8.0 Hz, 2H), 2.71 (t, *J*=7.1 Hz, 2H), 2.01 – 1.72 (m, 4H). **^{13}C NMR** (101 MHz, $CDCl_3$) δ 27.5, 28.1, 35.4, 68.4, 110.2, 114.8, 124.7, 125.1, 126.1, 127.5, 128.5, 141.8, 142.1, 149.6, 150.1. **MS** (ESI) *m/z*: 312 $[M + H]^+$, 329 $[M + NH_4]^+$, 350 $[M + K]^+$.

3-(6-Phenylhexanoyl)benzoxazol-2(3*H*)-one (9e)

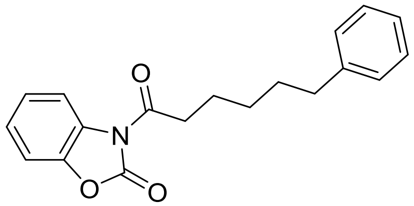

6-Phenylhexanoic acid **15** (0.67 mL, 3.60 mmol) was dissolved in DCM (20 mL) and $SOCl_2$ (0.31 mL, 4.26 mmol) was added slowly at rt, followed by DMF (0.11 mL, 1.43 mmol). The resulting mixture was heated to reflux (42 °C) and stirred for 3 h; then the solvent was evaporated to obtain the crude acyl chloride. The acid chloride was taken up in dry THF (2 mL) and added dropwise over 10 min to a solution of 3*H*-1,3-benzoxazol-2-one **10** (0.40 g, 3.0 mmol) and dry Et_3N (1.83 mL, 13 mmol) in dry THF (2 mL) cooled to 0°C. The reaction mixture was heated to reflux (66 °C) for 2 h, followed by quenching in ice-water (50

mL). Sat. aq. NH_4Cl (25 mL) was added, and the two phases were separated. The aqueous layer was extracted with EtOAc (3 × 50 mL) and the combined organic phase was dried over Na_2SO_4, filtered, concentrated and purified by silica gel column chromatography (cyclohexane:EtOAc 90:10 to 85:15) to afford **9e** as a white solid (849 mg, 76%). ^{1}H NMR (400 MHz, $CDCl_3$) δ 8.19 – 7.99 (m, 1H), 7.31 – 7.21 (m, 4H), 7.21 – 7.14 (m, 4H), 3.11 (t, *J*=7.4 Hz, 2H), 2.65 (t, *J*=7.7 Hz, 2H), 1.87 – 1.77 (m, 2H), 1.75 – 1.65 (m, 2H), 1.53 – 1.43 (m, 2H). **^{13}C NMR** (101 MHz, $CDCl_3$) δ 172.7, 151.5, 142.6, 142.4, 128.5, 128.4, 128.0, 125.8, 125.4, 124.9, 116.1, 110.0, 36.8, 35.9, 31.3, 28.8, 23.9. **MS** (ESI) *m/z*: 308 $[M – H]^-$.

6-Bromo-2-oxo-*N*-(4-phenylbutyl)benzoxazole-3(2*H*)-carboxamide (16a)

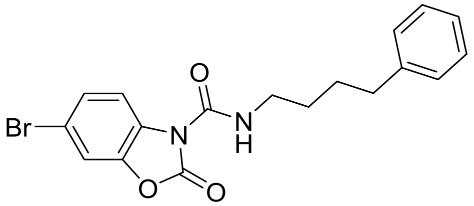

Compound **16a** was obtained according to the procedure previously described for **9a**, starting from 6-bromo-3*H*-1,3-benzoxazol-2-one **18** (100 mg, 0.47 mmol) and 4-phenylbutyl isocyanate **11** (0.09 mL, 0.51 mmol). The crude was purified by silica gel column chromatography (cyclohexane:EtOAc 85:15) to afford **16a** as a white solid (149 mg, 82%). **^{1}H NMR** (400 MHz, $CDCl_3$) δ 8.00 – 7.96 (m, 1H), 7.95 (d, *J*=9.0 Hz, 1H), 7.43 – 7.38 (m, 2H), 7.31 – 7.27 (m, 2H), 7.21 – 7.15 (m, 3H), 3.55 – 3.35 (m, 2H), 2.67 (t, *J*=7.2 Hz, 2H), 1.89 – 1.60 (m, 4H). **^{13}C NMR** (101 MHz, $CDCl_3$) δ 152.7, 149.5, 142.2, 142.0, 128.5, 128.2, 126.1, 117.2, 116.9, 113.6, 100.1, 40.3, 35.6, 29.1, 28.7. **MS** (ESI) *m/z*: 212 and 214 $[M – CONH(CH_2)_4Ph]^-$.

6-(4-Fluorophenyl)benzoxazol-2(3*H*)-one (19)

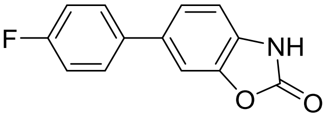

6-Bromo-3*H*-1,3-benzoxazol-2-one **18** (80 mg, 0.37 mmol) was dissolved in a 1:1 toluene/EtOH mixture (4 mL). (4-Fluorophenyl)boronic acid (78 mg, 0.56 mmol) was added followed by aq. Na_2CO_3 (2 M, 0.20 mL, 0.41 mmol). The resulting suspension was degassed under nitrogen for 10 min, followed by addition of $Pd(PPh_3)_4$ (43 mg, 0.04 mmol) and heating under microwave irradiation at 100 °C for 30 min. The reaction mixture was diluted with EtOAc (15 mL) and water (15 mL) was added. The two phases were separated and the aqueous layer was extracted with EtOAc (3 × 15 mL). The combined organic phase was dried over Na_2SO_4, filtered, concentrated and purified by silica gel column chromatography (cyclohexane:EtOAc 70:30) to afford **19** as a white powder (34.6 mg, 40%). ^{1}H NMR (400 MHz, $CDCl_3$): δ 7.08 (m, 3H), 7.33 (dd, *J* = 8.1, 1.6 Hz, 1H), 7.39 (d, *J* = 1.4 Hz, 1H), 7.47 – 7.52 (m, 2H), 7.91 (s, 1H). MS (ESI) *m/z*: 228 $[M – H]^-$.

6-(4-Fluorophenyl)-2-oxo-*N*-(4-phenylbutyl)benzoxazole-3(2*H*)-carboxamide (17a)

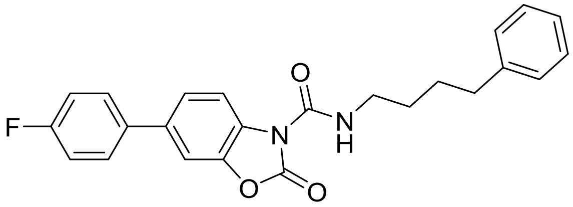

Compound **17a** was obtained according to the procedure previously described for **9a**, starting from 6-(4-fluorophenyl)benzoxazol-2(3*H*)-one **19** (35 mg, 0.15 mmol) and 4-phenylbutyl isocyanate **11** (0.03 mL, 0.16 mmol). The crude was purified by silica gel column chromatography (cyclohexane:EtOAc 87:13 to 80:20) to afford **17a** as a white powder (51 mg, 83%). **^{1}H NMR** (400 MHz, $CDCl_3$) δ 8.10 (d, *J*=8.3 Hz, 1H), 8.05 (t, *J*=5.4 Hz, 1H), 7.57 – 7.49 (m, 2H), 7.44 (dd, *J*=8.3, 1.6 Hz, 1H), 7.40 (d, *J*=1.6 Hz, 1H), 7.32 – 7.26 (m, 2H), 7.22 – 7.17 (m, 3H), 7.17 – 7.11 (m, 2H), 3.46 (td, *J*=6.7, 5.4 Hz, 2H), 2.68 (t, *J*=7.2 Hz, 2H), 1.83 – 1.64 (m, 4H). ^{13}C NMR (101 MHz, $CDCl_3$) δ 160.2 (d, *J*=285.3 Hz), 153.3, 149.9, 142.4, 142.0, 137.6, 128.9 (d, *J*=8.1 Hz), 128.6, 128.5, 127.3, 126.0, 123.9, 116.1, 115.9 (d, *J*=6.8 Hz), 108.6, 40.3, 35.6, 29.2, 28.7. **MS** (ESI) *m/z*: 405 $[M + H]^+$.

3. Synthesis and Characterization of the Fluorogenic Probe

***N*-[(1S,2R)-2-hydroxy-1-(hydroxymethyl)-4-(2-oxochromen-7-yl)oxy-butyl]dodecanamide**

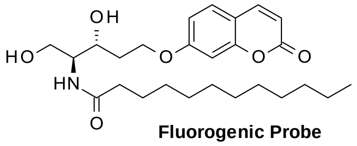

Fluorogenic Probe

The fluorogenic probe was synthesized by the improved method described by Xia Z et al.,[1] except for the last step (i.e. the acylation of the coumarinic aminodiol with dodecanoic acid), which was carried out as described by Bedia C et al,[2] using EDC·HCl, 1-hydroxybenzotriazole, and *N*,*N*-diisopropylethylamine in DMF. Analytical data of the fluorogenic probe:

White solid. Purity > 95%; **^{1}H NMR** (400 MHz, DMSO-d_6) δ 8.01 (d, *J*=9.5 Hz, 1H), 7.64 (d, *J*=8.4 Hz, 1H), 7.54 (d, *J*=8.4 Hz, 1H), 6.96 (m, 1H), 6.93 (d, *J*=2.4 Hz, 1H), 6.30 (d, *J*=9.5 Hz, 1H), 4.84 (d, *J*=6.2 Hz, 1H), 4.54 (t, *J*=5.5 Hz, 1H), 4.19 (t, *J*=6.6 Hz, 2H), 3.76 – 3.64 (m, 2H), 3.57 – 3.51 (m, 2H), 2.17 – 2.04 (m, 2H), 2.02 – 1.92 (m, 1H), 1.76 – 1.66 (m, 1H), 1.54 – 1.41 (m, 2H), 1.26 – 1.19 (m, 16H), 0.85 (t, *J*=7.1 Hz, 3H). **^{13}C NMR** (101 MHz, $CDCl_3$) δ 172.2, 162.0, 160.2, 155.4, 144.3, 129.4, 112.6, 112.31, 112.2, 101.1, 66.7, 65.6, 60.6, 55.2, 35.5, 32.8, 31.3, 29.0, 28.9, 28.8, 28.7, 28.6, 25.3, 22.0, 13.9. **MS** (ESI) *m/z*: 462.2 $[M + H]^+$. **$[\alpha]^{20}_D$** = -10.13 ± 0.6 (*c* = 0.43, $CHCl_3$).

4. Summary of AC Inhibitory Activity Data for Literature Compounds (1-7)

Table S1. AC inhibitory activity data and assay methods for compounds **1**-**7**.

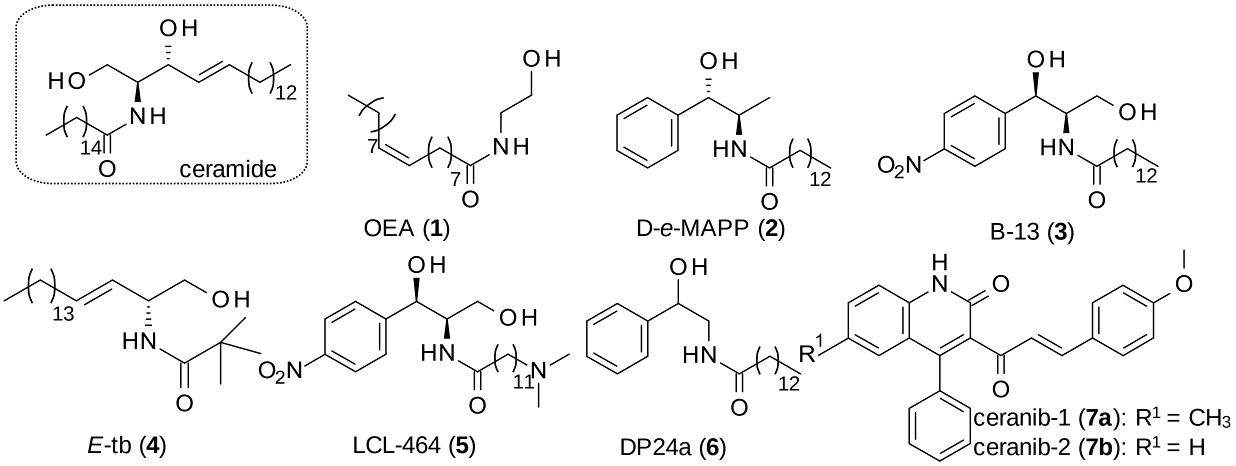

Compound	AC Inhibitory Activity	Assay	Protein Source	References
1	$IC_{50} > 500\ \mu M$	Radiometric	HL60 cells	Bielawaska et al., JBC **1996**
2	$IC_{50} > 500\ \mu M$	Radiometric	HL60 and HaCAT cells	Bielawaska et al., JBC **1996**; Raisova et al., FEBS **2002**
3	$IC_{50} \approx 10\ \mu M$	Radiometric	HaCAT cells	Raisova et al., FEBS **2002**
4	$IC_{50} \approx 15\ \mu M$	Fluorogenic	AC overexpressing cells	Bedia et al., ChemPhys of Lipids, **2008**
5	70% Inh.@50 μM	Radiometric	MCF7 cells	Bai et al., BioorMedChem Lett., **2009**
6	$IC_{50} = 1.287\ \mu M$	Micellar	Purified AC	Proksch et al., J.of Lipids **2011**
7	$IC_{50} = 28\ \mu M$	Fluorogenic	SKOV3 cells	Draper et al. *Mol Cancer Ther*, **2011**

5. Biology – Materials and Methods

Cell culture and treatments

SW403 and Raw 264.7 cells were purchased from American Type Culture Collection (Manassas, VA) and cultured in Dulbecco's Modified Eagle's Medium (DMEM) containing 10% fetal bovine serum at 37 °C and 5% CO_2. Drugs were dissolved in DMSO (10mM) and diluted in cell media (DMSO 0.4%) for cell treatments.

Animals and treatments

Male C57BL/6 mice (20–35 g, Charles River) were group-housed at room temperature on a 12 h light/dark cycle. Water and standard chow pellets were freely available. Drugs were dissolved in 15% polyethylene glycol, 15% Tween-80 and 70% saline (injection volume, 1mL/kg; i.p.). Animals were sacrificed 3 h after drug administration; tissues were collected, frozen in liquid nitrogen and stored at - 80 °C.

All procedures were performed in accordance with the Italian regulations on the protection of animals used for experimental and other scientific purposes (D.M. 116192), and European Economic Community regulations (O.J. of E.C. L 358/1 12/18/1986).

Preparation of enzyme-enriched lysate

Cells (10^6 cells) or tissues (10-20mg) were suspended in 20 mM Tris HCl (pH 7.5) with 0.32 M sucrose, sonicated and centrifuged at 800*g* for 15 min at 4 °C. Supernatants were then centrifuged at 12.000*g* for 30 min at 4 °C. Pellets were re-suspended in PBS buffer (pH 7.4) and subjected to two freeze-thaw cycles at - 80 °C. The suspension was finally centrifuged at 105.000*g* for 1 h at 4 °C and protein concentration was measured in the supernatant with bicinchoninic acid based protein assay.

Acid ceramidase (AC) activity: fluorescence-based *in vitro* assay

Screening of compounds and IC_{50} value determinations towards AC were performed by an optimized fluorescence-based *in vitro* assay based on principles described in literature.[3] Lysosomal lysate, enriched with AC, was prepared from a Hek293 cells stably expressing human AC. The assay was performed in Optiplate 96-wells black plates, with each reaction well containing a mixture of 25 mM sodium acetate buffer (pH 4.5) and a fixed amount of protein (2 µg) in a volume of 85 µL. After 30 min of pre-incubation with test compounds (diluted 20x from DMSO stock solutions at different concentrations), the fluorogenic probe was added (diluted 20x from EtOH stock solution, final concentration 5 µM). After incubation for 3 h at 37 °C, the reactions were stopped with 50 µL of MeOH and 100 µL of a 2.5 mg/mL $NaIO_4$ fresh solution in 100 mM glycine/NaOH pH 10.6. The plate was incubated at 37 °C for 2 h in the dark and fluorescence intensities were measured at excitation/emission wavelengths of 360/446 nm. Negative control samples consisted of the same incubation mixture in the absence of protein extracts. IC_{50} values of compounds were generated by quantifying the generation of umbelliferone as a function of compound concentrations**.** IC_{50} values were calculated by non-linear regression analysis of log[concentration]/inhibition curves using GraphPad Prism 5 (GraphPad Software Inc., CA – USA) applying a standard slope curve fitting.

Acid Ceramidase (AC) activity: LC-MS-based assay

AC activity in cells and tissues were measured with LC-MS based assay. Total lysates from cells or lysosomal preparations from tissue were diluted in assay buffer (100 mM sodium phosphate, 0.1% Nonidet P-40, 150 mM NaCl, 3 mM DTT, 100 mM sodium citrate, pH 4.5). Reactions were started by the

addition of 50 µM *N*-lauroyl ceramide (Nu-Chek Prep, Elysian, MN) and carried on for 1 h at 37 °C. Reactions were stopped by addition of a mixture of chloroform/MeOH (2:1) containing 1 nmol 11-lauroleic acid (NuChek Prep). The organic phases were collected, dried under nitrogen and analyzed by UPLC-MS (Acquity, Waters) in the negative-ion mode monitoring the reaction product (lauric acid, *m/z:* 199) using 11-lauroleic acid as internal standard. Lipids were eluted on an Acquity UPLC BEH C18 column (50mm length, 2.1 mm i.d., 1.7 µm pore size, Waters) column at 0.5 mL-min^{-1} for 1.5 min with a gradient of MeCN and water, both containing 0.25% acetic acid and 5 mM ammonium acetate (70% to 100% MeCN in 0.5 min, 100% MeCN for 0.5 min, 70% MeCN for 0.4 min). The column temperature was 40 °C. Electrospray ionization (ESI) was in the negative mode, capillary voltage was 1 kV and cone voltage was 50 V. N_2 was used as drying gas at a flow rate of 500 L/h and at a temperature of 400 °C. The $[M\text{-}H]^-$ ion was monitored in the selected-ion monitoring mode (*m/z* values: lauric acid 199, 11-lauroleic acid 197.35). Calibration curves were generated with authentic lauric acid (Nu Check Prep).

Lipid extraction and ceramide analysis

Lipids were extracted with a chloroform/MeOH mixture (2:1, 3 mL) containing internal standards. The organic phase was collected, dried under nitrogen, and dissolved in chloroform/MeOH (1:3) for LC-MS analyses. Ceramides and sphingosine were analyzed by LC-MS/MS, using a Waters Acquity UPLC coupled with a Waters Xevo TQMS and interfaced with ESI. Separation was done on a Waters Acquity BEH C18 1.7 µm column (2.1 × 50 mm) at 60 °C. A step gradient of 0.1% formic acid in MeCN/water (20:80) as solvent A and 0.1% formic acid in MeCN/isopropyl alcohol (20:80) as solvent B was applied at a flow rate of 0.4 mL/min. Detection was in the positive ion mode. Capillary voltage was 3.5 kV and cone voltage was 25 V. The source temperature and desolvation temperatures were set at 120 °C and 600 °C respectively. Desolvation gas and cone gas (N_2) flow were 800 and 20 L/h, respectively.

Ceramides were identified by comparison of their LC retention times and MS/MS fragmentation patterns with those of authentic standards (Avanti Polar Lipids). Extracted ion chromatograms were used to quantify myristoyl ceramide (C14:0, *m/z*: 492.5 > 264.3), palmitoyl ceramide (C16:0, m/z 520.3 > 264.3), stearoyl ceramide (C18:0 *m/z*: 548.3 > 264.3), lignoceroyl ceramide (C24:0 *m/z*: 632.3 > 264.3), nervonoyl ceramide (C24:1 *m/z*: 630.3 > 264.3) and using lauroyl ceramide standard (*m/z*: 464.5 > 264.3). Detection and analysis were controlled by Waters MassLynx software version 4.1.

Sphingosine was identified by comparison of its LC retention times and MS^2 fragmentation patterns with those of authentic standards (Avanti Polar Lipids). Extracted ion chromatograms were used to quantify sphingosine standard (d18:1, *m/z*: 300.5 > 282.5). Detection and analysis were controlled by Waters MassLynx software version 4.1. Calibration curves were prepared for every experiment.

Statistics

GraphPad Prism software (GraphPad Software, Inc., USA) was used for statistical analysis. Data were analyzed using the Student t-test or 1-way ANOVA followed by Bonferroni post hoc test for multiple

comparisons. Differences between groups were considered statistically significant at values of $p < 0.05$. Results are expressed as mean ± S.E.M.

6. Human acid ceramidase (AC) expression and purification

Human AC variant 1 coding sequence (NM_177924) was purchased from Open Biosystems (clone ID 3923451), modified by the insertion of a 6xHis tag at the C-terminus and a Kozak consensus sequence at the *N*-terminus and subcloned in the mammalian expression vector pCDNA3.1, containing the neomycin resistance gene. Hek293 wild-type cells were transfected with human ACpCDNA3.1 construct.

A stable cell line of human AC-overexpressing Hek293 was generated by selecting cell clones with G418 (1 mg/mL) for human AC expression.

The selected human AC-overexpressing Hek293 clone was expanded and grown in Hek293 Freestyle Expression Medium (Invitrogen) supplemented with 10mM NH_4Cl in order to improve protein secretion.

Purification of human AC was performed from the cell supernatant, which was initially concentrated using an Amicon ultrafiltration stirred cell (Merck Millipore). The concentrated supernatant was subjected to a two-step (40 and 90%) ammonium sulfate precipitation. The pellet was then re-suspended, dialyzed against 20 mm Tris-HCl, 500mm NaCl, pH 7.2 and incubated with 1% sodium deoxycholate in order to remove histones and DNA. After incubation the protein solution was purified through a nichel-affinity chromatography (HisTrap HP, GE Healthcare). The final purification step was performed through a gel filtration chromatography (Superdex 200 10/300GL, GE Healthcare). The purified protein was stored at 4 °C in 20 mm Tris-HCl, pH 7.2, 10 mm DTT. Protein concentration was estimated using the extinction coefficient calculated from the protein sequence $\epsilon 280 = 77850\ m^{-1}\ cm^{-1}$. The pure recombinant protein yield was about 1 mg/L of cells supernatant.

7. Liquid Chromatography-Mass Spectrometry (LC-MS) Study

Recombinant purified human AC was initially incubated for 1 h at pH 4.5 (20 mM acetate buffer) in the presence of 3 mM DTT at 37 °C for protein activation. The addition of compound **9a** in a 1:10 (protein:compound) molar ratio was followed by an additional hour of incubation at 37 °C. After incubation, reaction was stopped adding cold (4 °C) acetone to the samples (10x in volume). The tubes were vortexed and centrifuged for 10 min at 5000*g* at 4 °C. The supernatant was then discarded and the resulting pellet was dried under nitrogen stream and suspended in 50 mM $(NH_4)_2CO_3$ pH 8 for tryspin digestion. Proteomic grade trypsin (Sigma Aldrich, Italy) was then added in 1:50 w/w ratio with the protein. After an overnight incubation at 37 °C, the resulting peptides were analyzed on a UPLC chromatographic system equipped with a BEH C18 reversed phase column (1 × 100 mm). Peptides were eluted with a linear gradient of MeCN in water (both added with 0.1% formic acid) from 3 to 50% in 8 min. Flow rate was set to 0.09 mL per minute. Eluted peptides were analyzed in positive ion mode by high resolution tandem mass spectrometry on a Synapt G2 qTOF mass spectrometer (UPLC, column and qTOF instrument were purchased from Waters, Milford MA, USA). A linear ramp of the collision energy from 15 to 45 eV was used to induce backbone fragmentation of the eluting peptides.

8. Analytical Stability Data (stability at pH 4.5, pH 7.4 and in *m*-plasma)

Stability in AC buffer and PBS: compounds were incubated at 10 μM concentration in AC assay buffer (pH 4.5) or in PBS buffer (pH 7.4). Both buffers were pre-heated at 37 °C. Compounds were sampled at time points. Final DMSO was 1%. Solutions were kept at 37 °C under shaking.

m-Plasma stability: compounds were incubated at 10 μM concentration in the buffer pre-heated at 37 °C and sampled at time points. Compound **16a** was tested at 20 μM, due to its very poor LC-MS response. Final DMSO was 1%. Solutions were kept at 37 °C under shaking. At time points a sample aliquot (50 μL) was collected and crashed in cold MeCN (200 μL). After centrifugation (10 min at 5000*g*), the supernatant was collected for LC-MS analysis.

Both plasma and buffer stability samples were analyzed on the same Xevo triple-quad UPLC system described above and eluted from a BEH C18 reversed phase column with a linear gradient of MeCN in water. Compound stability was evaluated from the corresponding MRM (multiple reaction monitoring) peak areas plotted versus time. The corresponding decay profile was fitted with Prism to derive the corresponding half-life values.

9. Mouse Liver Microsomal Stability

Compounds were pre-incubated with microsomes in 100 mM TRIS buffer pH 7.4 for 15 min. At time zero, cofactors were added to start the reaction. The final incubation conditions for each sample were: 1.25 mg/mL mouse liver microsomes, 5 μM compound (final DMSO 0.1%), nicotinamide adenine dinucleotide phosphate 1 mM, glucose-6-phosphate 20 mM, $MgCl_2$ 2 mM, glucose-6-phosphate dehydrogenase 2Units. The mixture was kept at 37 °C under shaking. Aliquots (50 μL) were taken at time-points and crashed with 150 μL of MeCN spiked with 500 nM warfarin as internal standard. Reference incubation, with microsomes but without cofactors, was kept at 37 °C and sampled at the end of the timecourse. After vortexing and centrifugation, 3 μL of supernatant are analyzed by LC-MS/MS by multiple reaction monitoring (MRM) as described above.

10. Off-Target Pharmacology

Table S2. Inhibitory activity (% Inh. at 10 μM) of compound **17a** against a set of enzymes.[a]

		**% Inhibition of control values**
**Enzyme tested**	**Classification**	**Mean (value 1; value 2)**
$sPLA_2$ *(h)* (type V)	Phospholipase	1.1 (1.7; 0.5)
PLC	Phospholipase	-19.7 (-19.9; -19.4)
COX1 *(h)*	Cyclooxygenase	-6.1 (-10.4; -1.8)
COX2 *(h)*	Cyclooxygenase	6.7 (-3.2; 16.6)
5-lipoxygenase *(h)*	Lipoxygenase	15.2 (17.6; 12.9)
12-lipoxygenase *(h)*	Lipoxygenase	16.2 (11.6; 20.7)
15-lipoxygenase-2 *(h)* (recombinant)	Lipoxygenase	-33.3 (-23.2; -43.3)
BACE-1 *(h)* (β-secretase)	Aspartic protease	-13.3 (-13.8; -12.9)
cathepsin D *(h)*	Aspartic protease	**67.0 (67.1; 66.9)**
caspase-3 *(h)*	Cysteine protease	1.9 (2.6; 1.3)
caspase-8 *(h)*	Cysteine protease	-0.9 (-0.3; -1.5)
cathepsin B *(h)*	Cysteine protease	9.9 (1.7; 18)
cathepsin G *(h)*	Serine protease	16.6 (6.3; 27)
MMP-2 *(h)*	Metalloprotease	7.2 (7.3; 7.1)
MGL[b]	Lipase	17.1 (13.4; 18.9; 19.1)

[a] The screening was performed by Cerep at 10 μM concentration of **17a**. Compound enzyme inhibition effect was calculated as a % inhibition of control enzyme activity. Values are expressed as means of two determinations. [b] The screening was performed at University of California-Irvine (UCI), as described in King A.R. et al. *Chem. Biol.* **2009**, *16*(10), 1045-1052. *(h)*: human.

11. Pharmacokinetic Study

<u>*Administration:*</u> **17a** was administered intraperitoneally (i.p.) and intravenously (i.v.) to CD1 mice at 10 and 1 mg/kg dose, respectively. Vehicle was a PEG400/Tween80/saline solution at 10/10/80% in volume, respectively. Three animals per dose were treated. Blood samples were collected at 0, 15, 30, 60, 120, 240 and 480 min, sacrificing three animals per time-point. Control animals treated with vehicle were also included in the study.

<u>*Preparation of samples of **17a**, standard curve and QC sample*</u>: plasma was separated from blood by centrifugation for 15 min at 3500*g* a 4 °C, collected in an Eppendorf tube and subsequently frozen to - 80 °C. After 3 days, samples were thawed in an ice bath and after a short centrifugation transferred (50 μL) into a 96-deepwell plate and 150 μL of MeCN spiked with the internal standard (I.S.) at a final 500 nM concentration was added. **9a**, a close analogue of **17a** showing a very similar retention time in the experimental conditions (3.96 min compared to 3.91 min) was chosen for this purpose. After agitation (3 min), the plate was centrifuged at 3000*g* for 10 min at 4 °C. 80 μL of supernatant were transferred in a 350 μL 96-well plate and 80 μL of water was added. Standard **17a** was spiked in net solvent (PBS pH 7.4 with 10% MeCN) to prepare a calibration curve over the 1nM – 10μM range. Three quality controls samples were also prepared spiking blank rat plasma with **17a** to final 20, 200 and 2000 nM

concentrations. Calibrators and QCs were crashed with MeCN spiked with the I.S. as described for the plasma samples. All the liquid handling process was performed using a Hamilton workstation.

LC-MS analysis: **17a** plasma levels were monitored on a Xevo TQ UPLC-MS/MS system (Waters, USA), using the calibration curve and the internal standard (**9a**). Chromatography was carried out on Acquity BEH C18 column (2.1×100mm, 1.7 μm particle size, Waters, USA). Column and UPLC-MS system were purchased from Waters Inc. Milford, USA. Flow rate was set 0.4mL/min. Eluents were A = water and B = MeCN, both added with 10mM $(NH_4)_2CO_3$ buffered at pH 8. After 0.5 min at 50% B, a linear gradient of B was applied from 50 to 100% in 4 min then hold at 100% for 30 sec. After the gradient, the system was reconditioned at 50% B for one min. 10 μL of each sample prepared as described above were loaded on column. MS parameters: negative ion mode; capillary 2KV; cone 55V; source temperature 120 °C; cone gas 20L/Hr; desolvation gas 600 L/Hr; desolvation temperature 400 °C. The following MRM transitions were monitored: **17a**: *m/z* = 228 -> *m/z* = 228 at 10eV of collision energy; **9a** (I.S.): *m/z* = 210 -> *m/z* = 210 at 10eV collision energy. The time/concentration profiles measured with the above mentioned system were then analyzed using PK Solutions Excel application (Summit Research Service, USA) to derive the pharmacokinetic data reported in Table S2.

Table S3. Plasma pharmacokinetic (PK) parameters of **17a** after single intravenous (i.v.) and intraperitoneal (i.p.) administration in mice.[a]

Descriptive curve parameters:	Unit	i.v. (1mg/kg)	i.p. (10mg/kg)
C_{max} (obs)	ng/mL	628.0	1767.9
T_{max} (obs)	min	5.0	30.0
**Curve area calculations:**			
AUC(0-t) (obs area)	ng-min/mL	51201.8	310417.8
**Volume of distribution calculations:**			
V_d (area)/kg	mL/kg	1841.7	11602.0
**Clearance calculations:**			
CL (area)/kg	mL/min/kg	17.731	17.553
**Additional calculations:**			
Half-life from V_d and CL	min	72.0	458.0

[a] Maximum observed concentration (C_{max}); maximum time (T_{max}); cumulative area under curve (AUC) for experimental time points (0–8 h); distribution volume (V_d); systemic clearance (Cl) based on observed data points (0–8 h).

12. *Ex vivo* Quantification of Long-Chain Ceramides

Table S4. Effects of **17a** (10 mg/kg, 3 h) on long-chain ceramides levels in lungs.

	Vehicle			Cmpd 17a (10mg/kg)			
	**mean**	**S.E.M.**	**n**	**mean**	**S.E.M.**	**n**	**P value**
**Cer (d18:1/20:0)**	2.39	0.184	6	2.62	0.069	6	0.2701
**Cer (d18:1/22:0)**	8.08	0.584	6	8.52	0.216	6	0.4966
**Cer (d18:1/24:0)**	16.77	1.431	12	18.83	1.041	11	0.2659
**Cer (d18:1/24:1)**	35.19	2.592	12	38.01	3.143	11	0.4948

13. References

[1] Z. Xia, J. M. Draper, C. D. Smith, *Bioorg. Med. Chem.* **2010**, *18*, 1003-1009.
[2] C. Bedia, L. Camacho, J. L. Abad, G. Fabrias, T. Levade, *J. Lipid Res.* **2010**, *51*, 3542-3547.
[3] C. Bedia, J. Casas, V. Garcia, T. Levade, G. Fabrias, *ChemBioChem* **2007**, *8*, 642-648.
